# Genomic Sequence of Canine Papillomavirus 19

**DOI:** 10.1128/genomeA.01380-16

**Published:** 2016-12-08

**Authors:** Michael J. Tisza, Hang Yuan, Richard Schlegel, Christopher B. Buck

**Affiliations:** a Tumor Virus Molecular Biology Section, Laboratory of Cellular Oncology, National Cancer Institute, Bethesda, Maryland, USA; b Department of Pathology, Lombardi Comprehensive Cancer Center, Georgetown University Medical School, Washington, District of Columbia, USA

## Abstract

It is generally assumed that individual papillomas (warts) are caused by infection with individual papillomavirus types. Deep sequencing of virions extracted from a canine oral papilloma revealed the presence of canine papillomavirus 1 (CPV1), CPV2, and a novel canine papillomavirus, CPV19. This suggests that papillomas sometimes harbor multiple viral species.

## GENOME ANNOUNCEMENT

Papillomas (warts) are benign epithelial tumors that affect animals ranging from birds to humans. Warts are typically caused by infection with any of hundreds of currently known host-specific papillomaviruses (1). Papillomaviruses are small nonenveloped icosahedral viruses that carry a circular double-stranded DNA (dsDNA) genome decorated with host-derived histones. Recently, it has been suggested that individual warts can sometimes be simultaneously coinfected with multiple types of papillomaviruses (2).

In this study, three distinct canine papillomavirus types were observed in a sample of CsCl-purified virions extracted out of papillomatous oral tissue from a domestic dog (*Canis lupus familiaris*, breed pug). Prior analysis of this same sample led to the discovery of canine papillomavirus 2 (CPV2) (3). DNA was extracted from the virion stock, and a sequencing library was prepared using the Nextera DNA library preparation kit (Illumina). Sequencing was performed on a MiSeq sequencing system with paired-end 250-bp reads, yielding roughly nine million reads.

Reads were trimmed for quality, and adapter sequences were removed using CLC bio Genomics Workbench version 9 (Qiagen). Reads were then assembled using the CLC bio *de novo* assembly tool. Three contigs corresponding to full-length papillomavirus genomes were obtained and were identified by querying the BLAST nr database with BLASTn searches of entire contig sequences (4). Contig 1 (8,103 bp, 8.3 million reads) matched CPV2 with 100% identity (3). Contig 2 (8,596 bp, ~480,000 reads) matched canine oral papillomavirus with 100% identity (5). Contig 3 (7,942 bp, ~8,000 reads) showed CPV7 as the nearest match, with 77.1% identity at the nucleotide level in the L1 (major capsid protein) open reading frame (ORF) (6). The depth of coverage for contig 3 averaged 190 reads. According to Papillomavirus Episteme (PaVE) genotyping tools (1), contig 3 is a new canine papillomavirus type in the genus *Taupapillomavirus*. Because it appears to be the 19th known papillomavirus type of dogs, we suggest that the new canine papillomavirus type be assigned the number 19.

CPV19 has a typical genome size and organization for viruses in papillomavirus genus *Taupapillomavirus*. It contains all putative ORFs on the same coding strand, including E6, E7, E1, E2, E5, L2, L1, and the typical spliced ORFs of E1^E4 and E8^E2.

### Accession number(s).

The complete genomic sequence of CPV19 was deposited in GenBank. It has been assigned the accession number KX599536.
